# Effect of the cPKCγ-Ng Signaling System on Rapid Eye Movement Sleep Deprivation-Induced Learning and Memory Impairment in Rats

**DOI:** 10.3389/fpsyt.2021.763032

**Published:** 2021-10-29

**Authors:** Shu Xu, Yanbo Zhang, Zhiqing Xu, Luping Song

**Affiliations:** ^1^School of Rehabilitation, Capital Medical University, Neurorehabilitation Center, Beijing Boai Hospital, China Rehabilitation Research Center, Beijing, China; ^2^Department of Neurobiology, The Second Affiliation Hospital of Shandong First Medical University, Tai'an, China; ^3^Department of Neurobiology, Beijing Key Laboratory of Neural Regeneration and Repair, Capital Medical University, Beijing, China; ^4^Department of Rehabilitation Medicine, Shenzhen University General Hospital, Shenzhen, China

**Keywords:** REM sleep deprivation, learning and memory, cPKCγ, neurogranin, signaling

## Abstract

**Objective:** Rapid eye movement sleep deprivation (REM-SD) can cause a decline in learning and memory and lead to changes in behavior. Therefore, REM sleep plays a key role in processes that govern learning and memory. However, the mechanism underlying REM-SD-induced learning and memory impairment is unclear and the underlying molecular signaling still needs to be identified. In the present study, we investigated the role of the cPKCγ-Ng signaling pathway in REM-SD-induced learning and memory impairment.

**Method:** Sixty male rats were divided into Control, REM-SD, REM-SD+cPKCγ activator PMA, REM-SD+cPKCγ inhibitor H-7, and sleep revival (SR) groups. The Morris water maze was used to assess spatial learning and memory. Western blot analysis was used to detect cPKCγ total protein expression and membrane translocation levels, and Ng total protein expression and phosphorylation levels.

**Results:** The REM-SD group performed worse on the Morris water maze test than the control group. Western blot analysis showed that cPKCγ membrane translocation and Ng phosphorylation levels were significantly lower in the REM-SD group. SR following REM-SD restored learning and memory ability, cPKCγ transmembrane translocation, and Ng phosphorylation levels, but not to levels observed before REM-SD. PMA and H-7 significantly improved/disrupted task ability as well as cPKCγ transmembrane translocation and Ng phosphorylation levels in REM-SD rats.

**Conclusion:** The REM-SD induced learning and memory impairment in rats and may be associated with the cPKCγ-Ng signaling pathway. Specifically, activation of the cPKCγ-Ng signaling pathway may protect against REM-SD.

## Introduction

Sleep deprivation (SD) can cause changes in mood, learning, memory, and immune function, which can lead to physiological, psychological, and even behavioral changes. Although studies have confirmed that SD can cause obvious cognitive impairment (1–3), the underlying mechanisms remain unknown. Studies that focus on the molecular signaling pathways that govern this process will be important for the development of effective therapeutic interventions.

In 2007, the American Academy of Sleep Medicine classified sleep into non-rapid eye movement (NREM) stages 1, 2, and 3 and a rapid eye movement (REM) stage, with each stage being interconnected. Normally, people pass through 4–6 cycles of these sleep stages each night. Compared to a waking state, REM sleep is characterized by low-amplitude, mixed frequency signals as seen on electroencephalograms (EEG), rapid eyeball movement, markedly decreased muscle tone, and increased cerebral blood flow and oxygen consumption. REM-SD can cause a decline in memory and cognitive functions. Therefore, REM sleep plays a key role in learning and memory (4–7). Neuroimaging is a powerful tool to explore the neural mechanism of sleep disorders. Studies have found that some certain diseases, such as optic neuritis, sleep deprivation, etc., can cause changes in the topology of the brain network, which may cause symptoms of cognitive impairment (8). However, it is also of great significance to carry out molecular signal mechanism research. Through the study of molecular signal targets, it may be possible to provide a new perspective for future drug interventions.

Protein kinase C (PKC) was first discovered by Nishizuka in 1977. PKC is a family of serine/threonine kinases that is widely expressed in many bodily systems and is an important intracellular signal transduction molecule. To date, at least 12 subtypes of PKC have been isolated and identified through molecular cloning and enzyme analysis, and these are widely distributed in various tissues and organs. PKC plays an important physiological role in the regulation of neuronal excitability, signal transduction, release of neurotransmitters, synaptic plasticity, learning and memory, cell proliferation, gene expression, cell degeneration, and programmed cell death (9, 10). Under resting conditions, PKC exists in the cytoplasm in an inactive form. When external stimulus exists, PKC is translocated to the cell membrane where it can exert its physiological role (11, 12). There is increasing evidence that PKC also plays an important role in spatial learning and memory (). Inhibition of PKC activity can impair learning and memory abilities, such as that induced by vascular dementia and traumatic brain injury (13–16). PKC activators have also been shown to have memory-enhancing and anti-dementia effects (17). Studies have shown that PKC plays an important role in changes that occur during SD; PKC mRNA levels in the hippocampus and pre-frontal cortex of rats were significantly reduced after periods of SD (18). However, PKC is considered to exert its physiological activities through its translocation, and changes in PKC activity during SD have not been studied, nor has anyone explored the roles that PKC subtypes play in SD-induced learning and memory impairment. cPKCγ is a classical PKC subtype that only exists in neurons of the brain and spinal cord. cPKCγ can regulate synaptic development and plasticity, and can directly affect long-term potentiation and long-term depression (LTD). Thus, cPKCγ has attracted increasing attention. Although some reports have found that cPKCγ is involved in learning and memory (19, 20), the role of cPKCγ in REM-SD-induced learning and memory impairment has been rarely studied. Therefore, in the present study, we investigated the role of cPKCγ in REM-SD-induced learning and memory impairment, and observed changes in its activity in the brain. The results thus contribute to our understanding of the molecular mechanism that underlies REM-SD-induced memory impairment, which is of critical importance in the field of SD.

Neurogranin (Ng) is a newly discovered neuro-specific postsynaptic protein member of the calpacitin protein family that comprises 78 amino acids. Ng is distributed in specific brain regions, including the cerebral cortex, hippocampus, and olfactory bulb, and is a postsynaptic substrate of PKC (21). Ng can be phosphorylated by PKC and participates in the induction of long-term potentiation and long-term depression. Ng gene expression and protein synthesis are synchronized with synapse formation and neuronal differentiation, which indicates that Ng may be involved in physiological processes such as learning, memory, and development of the nervous system. Under certain pathophysiological conditions, such as hypothyroidism, SD, and hypoxic preconditioning, Ng activity plays roles in changing nervous system functions (22–26). One study found that SD resulted in a significant reduction in Ng mRNA levels in the rat hippocampus and pre-frontal cortex (18). Ng exerts its physiological activity after being phosphorylated by PKC, but changes in Ng activity after REM-SD have not been explored, nor have the subtypes of PKC that phosphorylate Ng in REM-SD-induced learning and memory impairment been identified.

Both PKC and Ng play important roles in learning and memory. In the present study, we investigated the role of the PKC-Ng signaling system in REM-SD-induced learning and memory impairment in rats. The key aim was to identify which subtypes of PKC phosphorylate Ng and play crucial roles in learning and memory impairment. Represa et al. found that Ng is widely distributed in the cerebral cortex, thalamus, and striatum, and is scarce in the cerebellum and brain stem, which is consistent with the distribution of cPKCγ in the brain (27). Sato et al. (28) further found that Ng and cPKCγ exhibit similar gene expression patterns during brain development. Neuner-Jehle et al. found that after 24 h of SD, mRNA and expression of Ng differed depending on the brain region. Ng mRNA was significantly reduced in the forebrain and midbrain regions, but did not change in the hippocampus. Ng protein levels were significantly reduced in the cortex, but did not change significantly in other brain regions (29). This suggests that it may be more meaningful to study the changes in the PKC-Ng signaling system in the pre-frontal cortex of animals. Given this theoretical basis, it is important to investigate the cPKCγ-Ng signaling system and its role in REM-SD-induced learning and memory impairment. In the present study, we established a rat model of 72-h REM-SD and 12-h sleep revival (SR), and identified changes in the cPKCγ-Ng signaling system in the frontal cortex of the rats. Additionally, we measured Ng phosphorylation levels after administration of the cPKCγ activator PMA and the cPKCγ inhibitor H-7 and explored the underlying molecular mechanisms.

## Materials and Methods

### Experimental Animals

**Figure 1:**
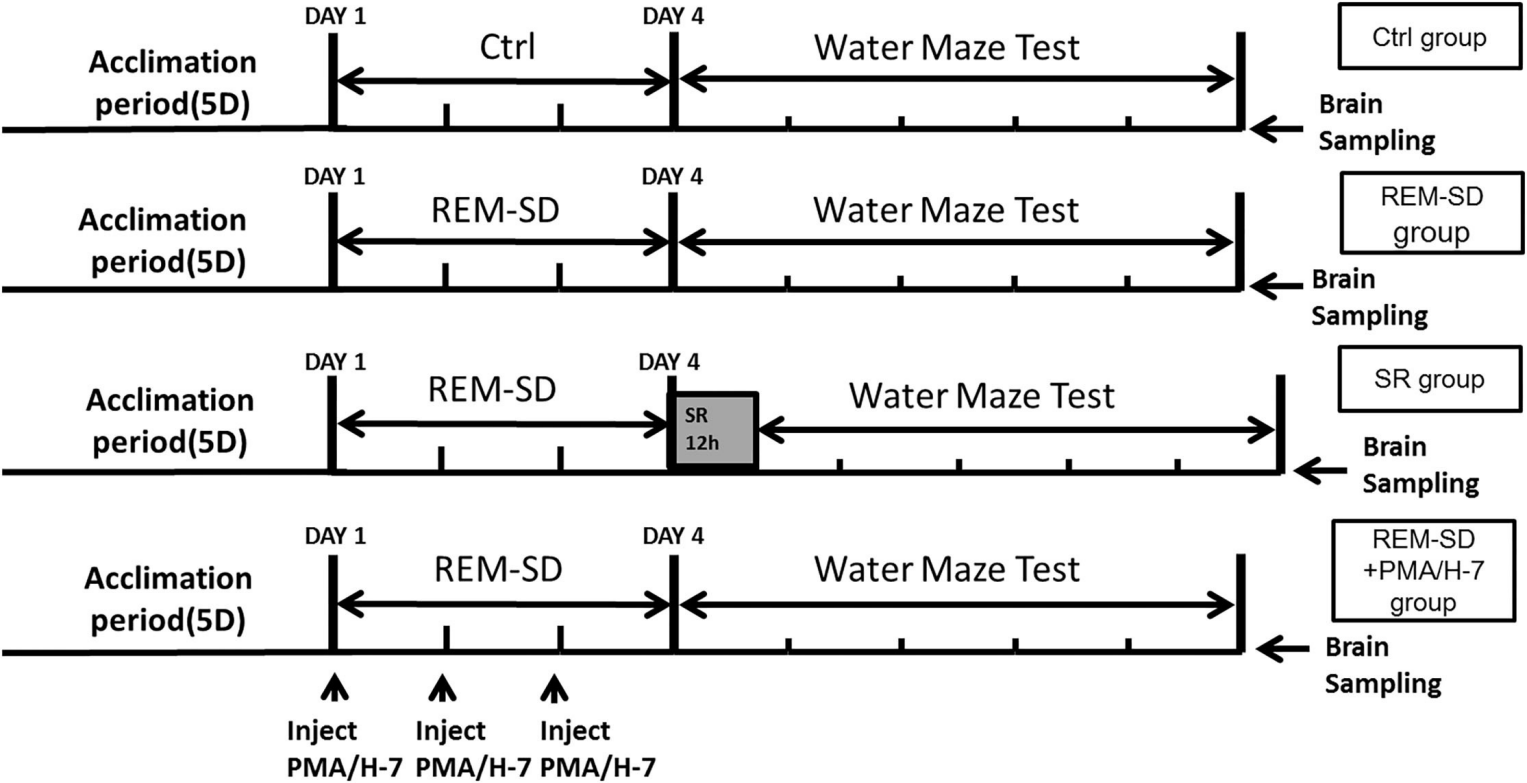
Overview of the experimental procedures.

Sixty male Sprague Dawley rats (15 weeks old) were used in the experiment [provided by the Experimental Animal Center, Shandong University of Traditional Chinese Medicine, China; License No.: SCXK (Lu) 20050015]. All rats were provided with standard food pellets and tap water and housed in a quiet room set to 18–25°C and 40–60% humidity. The rats were divided into five groups (12 rats/group). Control group: rats were housed in a normal rat cage with a natural 12/12 h light–dark cycle (lights on at 7:00 A.M.), and did not undergo REM-SD. REM-SD group: rats were continuously subjected to a fluorescent lamp for 72 h. After REM-SD was completed, the rats were taken out of the REM-SD box and then returned to their cages. REM-SD+PMA group: during the 72-h continuous REM-SD, 20 μl PMA solution (containing 100 ng PMA, phorbol-12-myristate-13-acetate C2230, Sigma) was injected into the spinal subarachnoid space once every 24 h (three injections overall). The dose of PMA was chosen according to a previous study (11) REM-SD+H-7 group: during the 72-h continuous REM-SD, 20 μl H-7 solution (containing 200 μg H-7, soluble in saline, I6891, Sigma) was injected into the spinal subarachnoid space once every 24 h (three injections overall). The dose of H-7 was chosen according to previous study (11). SR group: after the rats were subjected to 72 h of continuous REM-SD, rats were placed in a cage for 12 h of SR. An overview of the experimental procedure is shown in Figure 1.

### Catheterization of the Spinal Subarachnoid Space

Rats were anesthetized with sodium pentobarbital (40 mg/kg) intraperitoneally. The skin was incised at the midline of the occipital bone and separated layer by layer. Heads were tilted forward by about 30° and a polyethylene catheter (PE-10) was caudally inserted into the subarachnoid space. After surgery, rats were injected intramuscularly with gentamicin sulfate (40,000 units/rat/day) for 3–5 days. Normal rats without hemiparesis were used for the experiments.

### Establishment of the REM-SD Model

REM-SD was induced by the modified multiple platform method as described previously (30, 31). The modified multiple platform method is the most widely used technique mainly for deprivation of REM sleep, but not for deprivation of non-REM sleep (NREM). A self-made REM-SD box (110 × 60 × 40 cm) had 15 small platforms (6.5 cm in diameter and 8.0 cm in height) that were set up at 15-cm intervals. The box was filled with water, and the surface of the water was about 1.0 cm below the surface of the platforms. Water and food were placed on the top of platforms. Rats had free access to food and water and were allowed to move freely between platforms. Due to their instinctive fear of water, rats slept on the platforms and could enter NREM. However, if the rats entered REM sleep and lost their muscle tone, they would fall into the water and awaken, thus being prevented from entering REM sleep. This technique has been validated using electroencephalography and has been shown to eliminate ~90–95% of REM sleep (32).

### The Morris Water Maze

The Morris water maze is a widely used method to assess spatial learning and memory. Maze testing was carried out by the SMART-CS (Panlab, Barcelona, Spain). This maze comprised a circular pool with a 160 cm-diameter and a 55 cm-high wall. The pool was divided into four equal quadrants. Powdered milk was added to make the water opaque, and the water was kept at 22–25°C. A circular transparent platform (12 cm in diameter and 25 cm in height) was located in the center of the third quadrant, hidden 1 cm below the surface of the water. The Morris water maze test was conducted for 5 days, beginning immediately after 72-h REM-SD and 12-h SR, and the navigation probe test was performed within the first 4 days of the experiment. For each trial, a rat was put into the water from the midpoint of each quadrant facing the maze's wall. The rat was allowed to swim for 120 sec to find the hidden platform and climb onto it. If the rat found the platform within 120 sec, the rat was allowed to remain on the platform for 4 sec, then the trial was terminated and the latency was recorded; if the rat had not found the platform in 120 sec, it was guided and placed on the platform for 30 sec by the experimenter, the trial was terminated and latency period was recorded as 120 sec. There was an intertrial interval of 5 min. The position of rats in the pool was tracked using an overhead camera connected to a SMART-CS program. The escape latency, swimming distance, and swimming speed were recorded (average of four trials) to evaluate the spatial learning of rats.

The spatial probe test was performed on day 6 to evaluate the spatial memory of rats. In this test, the platform was removed, the rats were placed into the water from the first quadrant, and were allowed to swim for 120 sec. The time spent in the target quadrant (where the platform had previously been placed) and the other quadrants within 120 sec was recorded.

### Western Blot Analysis

The pre-frontal cortices of rats in each group were removed and frozen in liquid nitrogen and stored at −80°C for future use.

*Detection of cPKC*γ *protein expression in cytosolic and membrane fractions*: The frozen tissues were thawed and homogenized in Buffer A (10 μl/mg tissue, containing 50 mmol/L Tris-HCl pH 7.5, 2 mmol/L EDTA, 2 mmol/L EGTA, 2 mmol/L DTT, 5 mmol/L Napyrophosphate, 50 mmol/L KF, 5 × 10^−5^ mmol/L Okadaic acid, and 5 ug/ml each of leupeptin, aprotinin, pepstatin A, and chymostatin). After centrifugation at 30,000 g for 30 min at 4°C, the supernatant was collected as cytoplasmic fraction (cytosol) and stored. Then, Buffer B (Buffer A + 0.5% NP-40) was added to the remaining pellet, the procedure described above was performed, and the supernatant was collected as a membrane fraction (particulate). *Total cPKC*γ *protein*: The frozen tissues were thawed and homogenized in Lysis Buffer containing 50 mM Tris-HCl pH 7.5, 5 mM EDTA, 5 mM EGTA, 1.0% SDS, 150 mM NaCl (10 μl/mg tissue). *Ng protein*: The frozen tissues were thawed and homogenized in lysis buffer (10 μl/mg tissue) containing 50 mM Tris-HCl, pH 7.5, 2 mM EDTA, 2 mM EGTA, 2 mM DTT, 5 mM Na pyrophosphate, 50 mM KF, 50 nM Okadaic acid, 5 mg/ml each of leupeptin, aprotinin, pepstatin A, and chymostatin, and 1.0% SDS. Protein concentration was measured using a BCA kit (Pierce Company, Rockford, USA). SDS-PAGE loading buffer was added to the protein samples (3 μg/μL) and the samples were boiled for 5 min and then stored at −80°C for western blot analysis. Antibodies included cPKCγ rabbit polyclonal antibody (1:1000, Santa Cruz, Dallas, Texas), Ng mouse monoclonal antibody (1:1000, Santa Cruz, Dallas, Texas), phosphorylated Ng rabbit monoclonal antibody (1:1000, Merck-Millipore, ABN426), β-actin rabbit polyclonal antibody (1:1000, Santa Cruz, Dallas, Texas), β-actin mouse monoclonal antibody (1:1000, Santa Cruz, Dallas, Texas), Na-K ATPase monoclonal antibody (1:1000, Santa Cruz, Dallas, Texas), goat anti-rabbit secondary antibody (1:5000, Santa Cruz, Dallas, Texas), and goat anti-mouse secondary antibody (1:5000, Santa Cruz, Dallas, Texas). Western blots were developed using the SuperSignal West Pico Chemi-luminescent Substrate kit (Pierce Company, Rockford, USA). After membranes were exposed, the bands were scanned and analyzed using Quantity One 1-D analysis software (Bio-Rad Laboratory, Hercules, CA). The contents of cPKCγ cytoplasmic protein and membrane protein were calculated using β-actin as an internal reference. The cPKCγ membrane translocation level was expressed by the percentage of membrane protein content to total protein (membrane protein + cytoplasmic protein) in each group. The membrane translocation level of the control group was taken as 100%, and the cPKCγ membrane translocation level in each experimental group was expressed using a percentage. Taking β-actin as an internal reference, the total protein contents of cPKCγ and Ng were calculated for each group. The protein expression in the control group was 100%, and the change of protein expression in each group was expressed as a percentage. The Ng phosphorylation level was expressed as the ratio of Ng phosphorylated protein to total protein.

### Statistical Analysis

Results were analyzed using a two-way ANOVA and one-way ANOVA using SPSS 22.0 software. For significant results identified by the one-way ANOVA, Tukey's *post hoc* tests were used to compare the differences between multiple groups. A value of *P* < 0.05 was considered statistically significant.

## Results

### REM-SD- Induced Behavioral and Physiological Changes in Rats

As the length of sleep deprivation increased, behavioral and physiological changes in rats became increasingly obvious. The primary manifestations included increased lassitude, dullness, lack of luster, dryness, disheveled and untidy fur, increased excitability and irritability, and increased alertness, responsiveness, and aggressive behavior in response to environmental stimuli. Some rats even suffered from mucosal bleeding of the paws due to scratching. These symptoms indicated successful establishment of the rat model of REM-SD.

### REM-SD Induced Changes of Morris Water Maze Test in Rats

#### REM-SD Prolongs the Average Escape Latency in Rats

**Figure 2:**
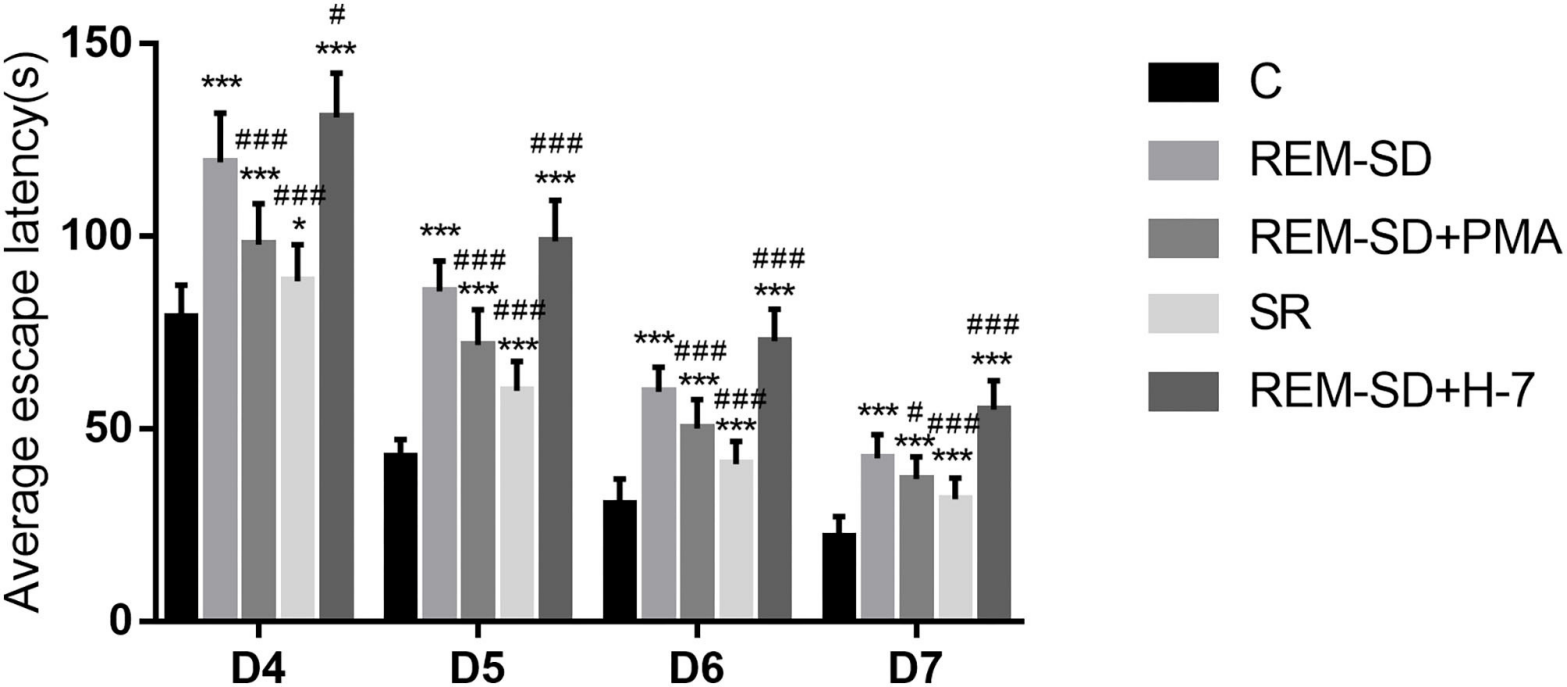
Average escape latency (seconds) for all groups. Compared with the control group (C), the average escape latencies for all other groups were significantly prolonged on each day of training. Compared with the REM-SD group, the average escape latency was significantly shorter in the SR and REM-SD+PMA groups, and was significantly longer in the REM-SD+H-7 group *P < 0.05, ^#^*P* < 0.05 vs. control group, ***^/*###*^means *P* < 0.001.

Results from the place navigation test showed that, for all groups, the average time needed to find the platform gradually shortened during training. However, compared with the control group, the average escape latencies for all other groups were significantly prolonged on each day of training. Compared with the REM-SD group, the average escape latency was significantly shorter in the SR and REM-SD+PMA groups, and was significantly longer in the REM-SD+H-7 group (Figure 2).

#### REM-SD Decreases the Percentage of Time Spent in the Target Quadrant

**Figure 3:**
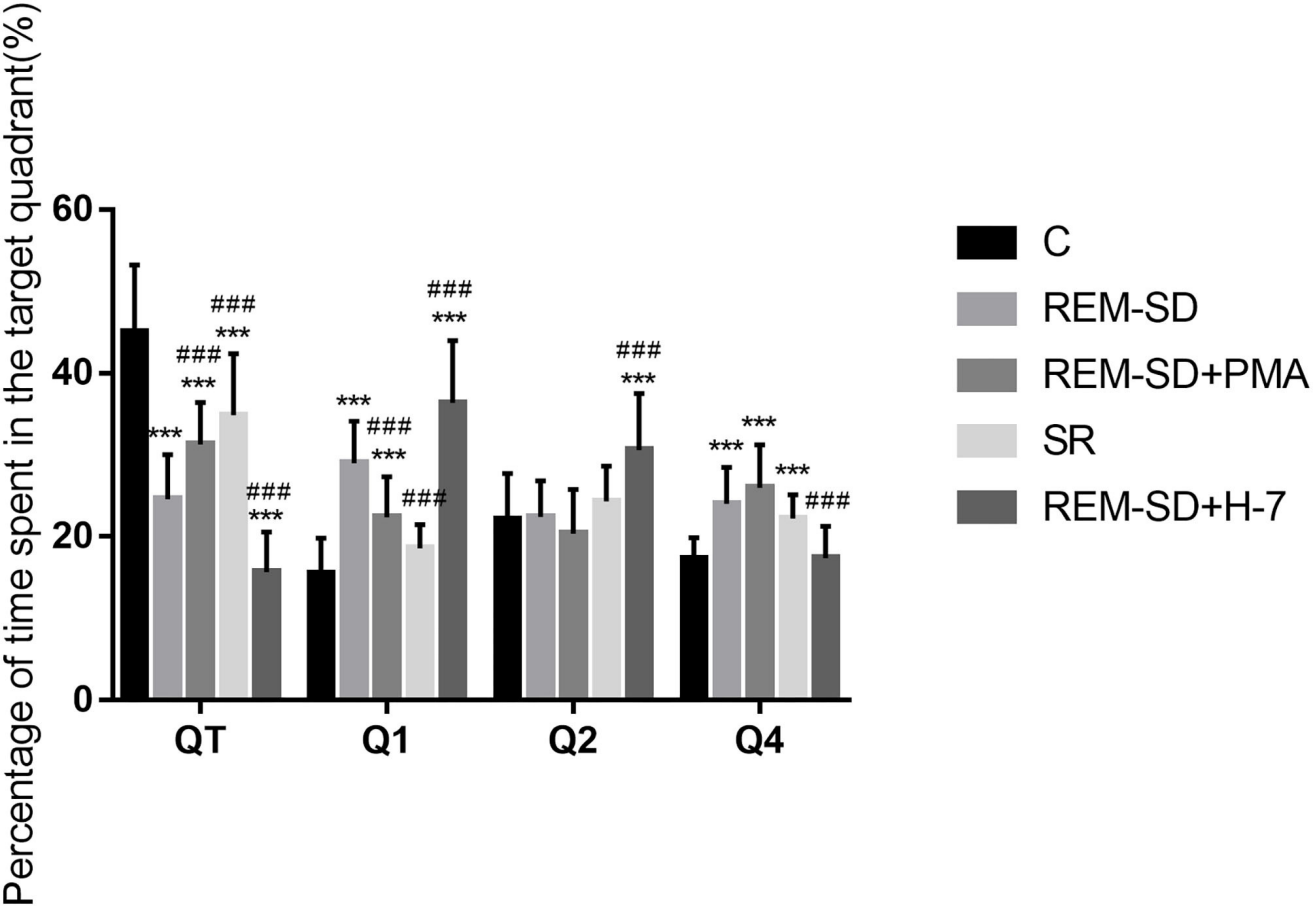
The percentage of time spent in target quadrant for each group. Compared with the control group (C), the percentage of time spent searching the target quadrant (where the platform was placed) was significantly lower in the REM-SD, REM-SD+PMA, REM-SD+H-7, and SR groups. Compared with the REM-SD group, this time was significantly higher in the SR and REM-SD+PMA groups and lower in the REM-SD+H-7 group. ***^###^means *P* < 0.001.

Results from the spatial probe test showed that, compared with the control group, the percentage of time spent on searching for the target quadrant (where the platform was placed) was significantly lower in the REM-SD, REM-SD+PMA, REM-SD+H-7, and SR groups. Compared with the REM-SD group, this time was significantly higher in the SR and REM-SD+PMA groups and lower in the REM-SD+H-7 group. The REM-SD, REM-SD+PMA, and REM-SD+H-7 groups spent significantly more time in the first quadrant than did the control group, and no significant difference was found between the SR and control groups. Compared with the REM-SD group, the percentage of time spent in the first quadrant was lower in the SR and REM-SD+PMA groups, and higher in the REM-SD+H-7 group (Figure 3).

#### REM -SD Does Not Affect Swimming Speed in Rats

**Figure 4:**
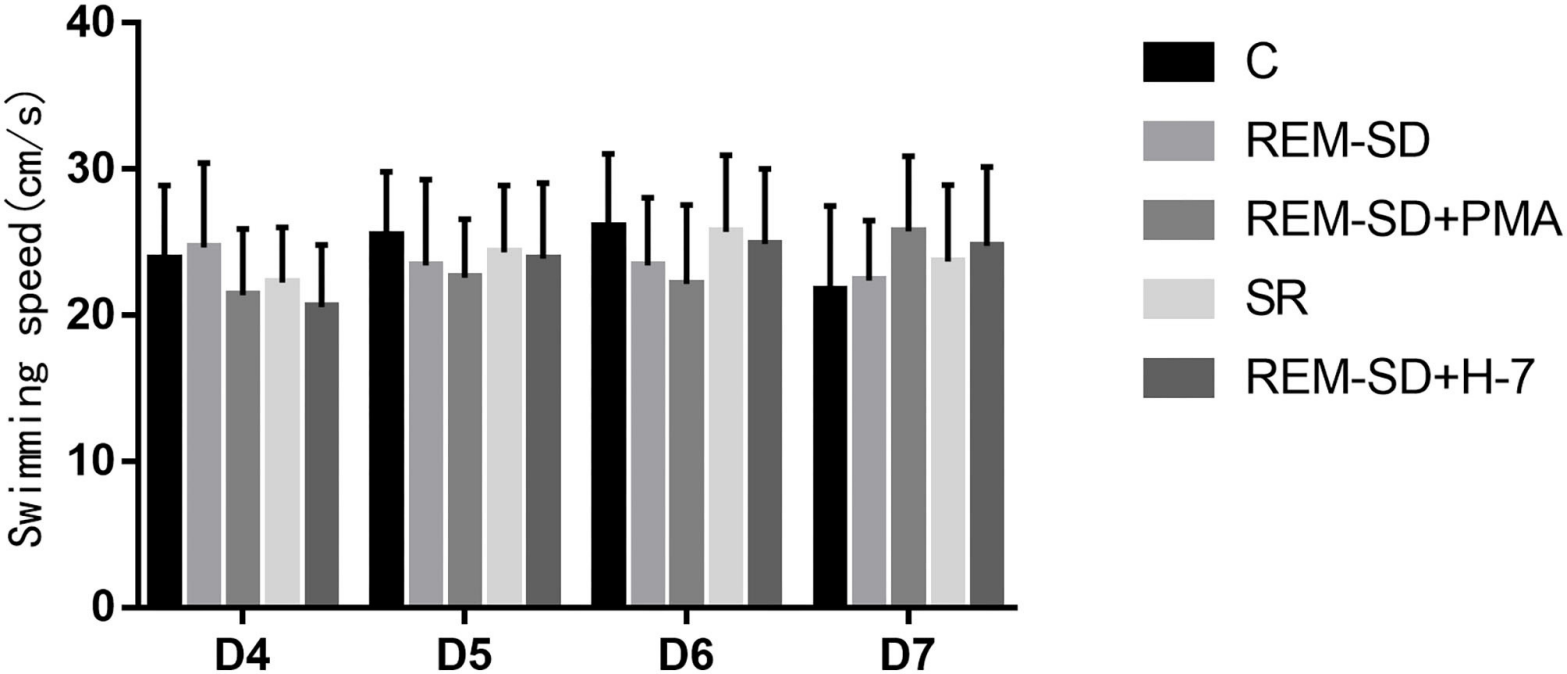
The average swim speed (cm/s) on the Morris water-maze test for each group. No significant difference was found between the five groups with regard to the average swimming speed on the Morris water-maze test.

Average swimming speed did not differ across the five groups (Figure 4) on the Morris water-maze test.

### REM-SD Reduces cPKCγ Protein Expression

**Figure 5:**
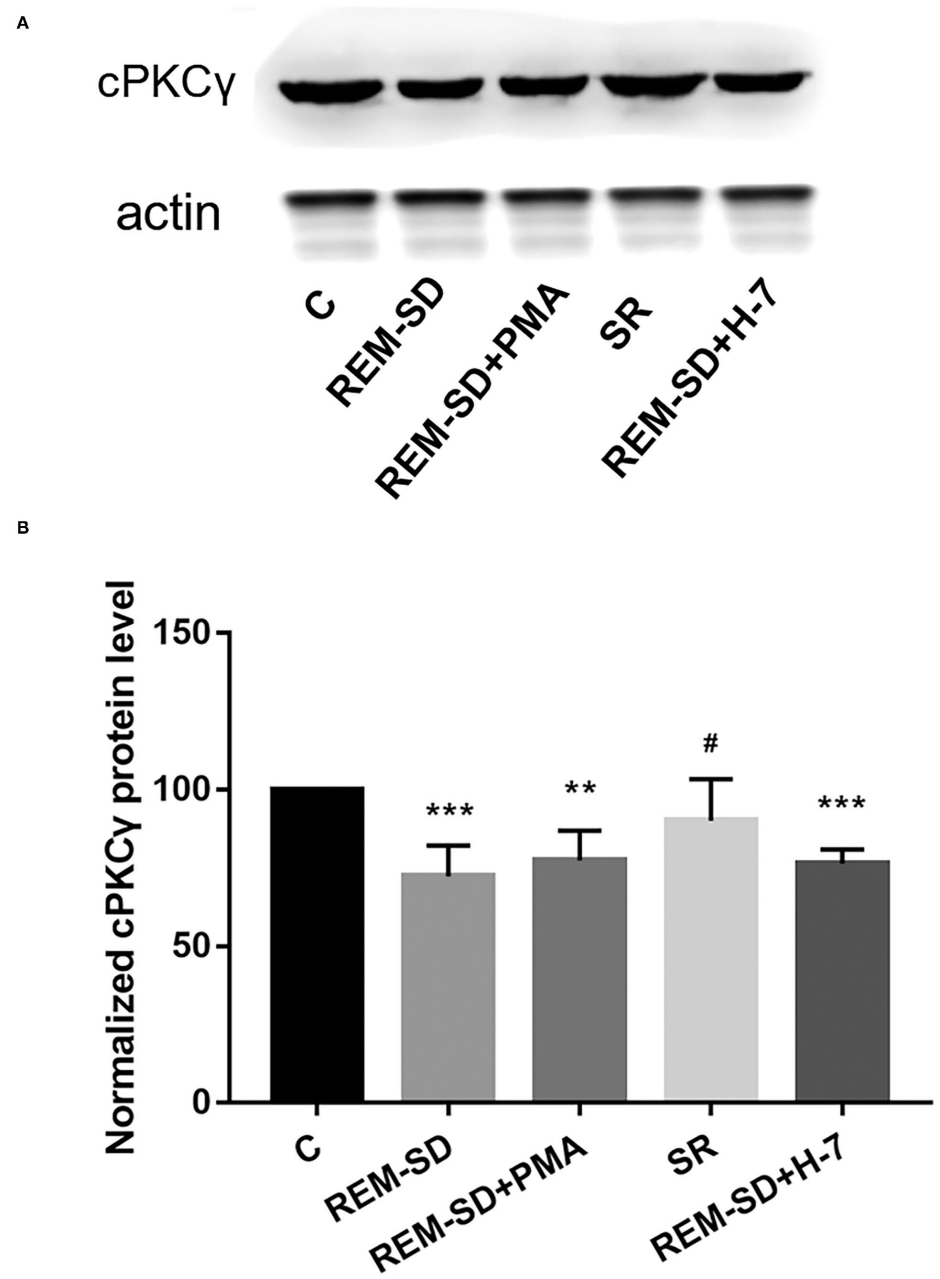
cPKCγ protein expression in the prefrontal cortex of SD rats from each group and control group (C). **(A)** Western blot analysis results. **(B)** The relative protein expression of cPKCγ for each group. **P < 0.01, ***P < 0.001 vs. control group; ^#^*P* < 0.05 vs. SD group.

Results from Western blot analysis showed that cPKCγ protein expression in the REM-SD, REM-SD+PMA, and REM-SD+H-7 groups was significantly lower than that in the control group. Levels did not differ between the SR and control groups, between the REM-SD and REM-SD+PMA groups, or between the REM-SD and REM-SD+H-7 groups. cPKCγ protein expression in the SR group was significantly higher than that in the REM-SD group (Figure 5).

### REM-SD Inhibits cPKCγ Membrane Translocation

**Figure 6:**
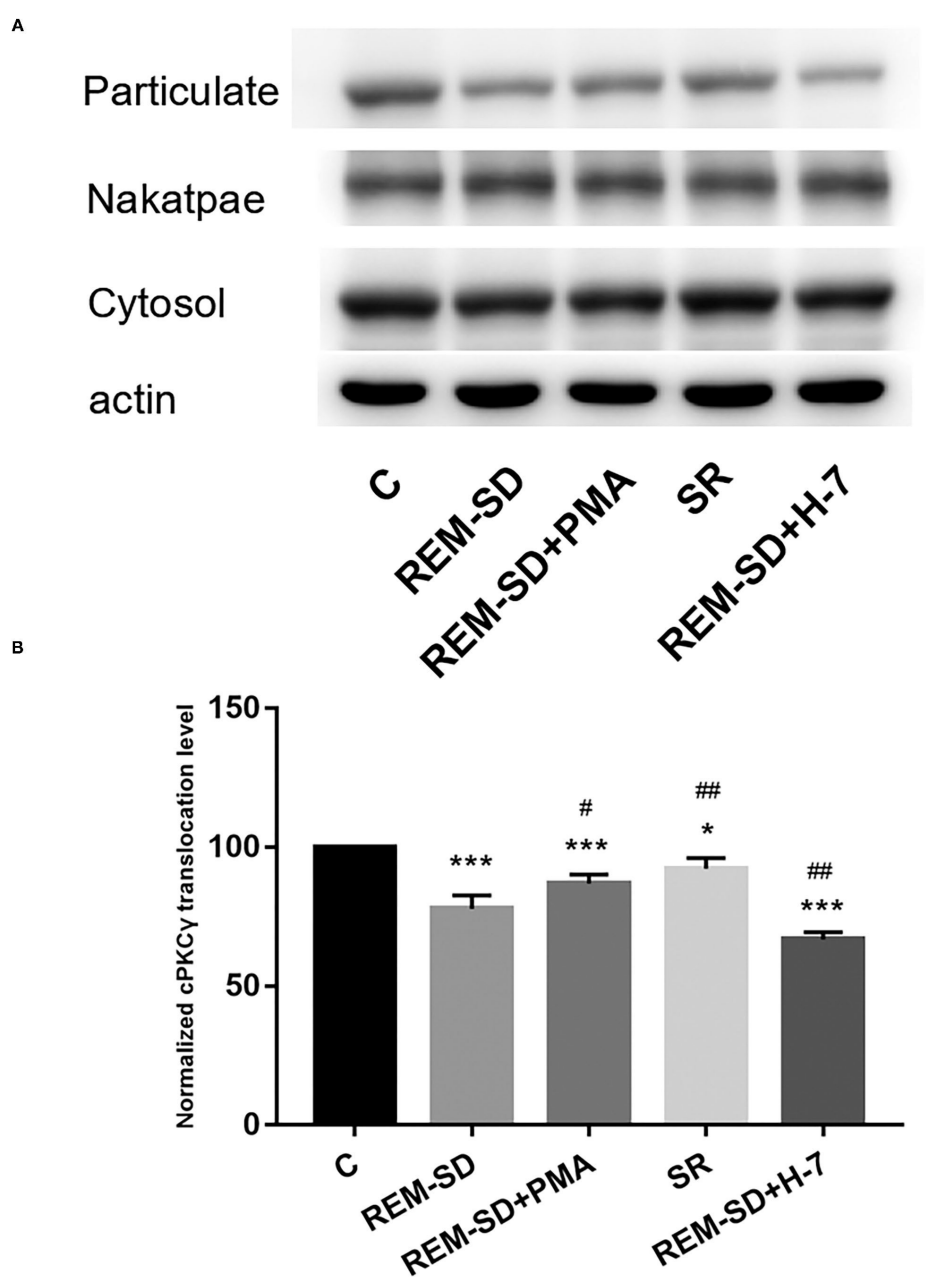
cPKCγ membrane translocation levels in the prefrontal cortex of SD rats from each group and control group (C). **(A)** Western blot analysis results. Cytosol and particulate-represented cytosolic and membrane proteins, respectively. **(B)** cPKCγ membrane translocation levels for each group. *P < 0.05, ***P < 0.001 vs. control; ^#^*P* < 0.05, ^##^*P* < 0.01, vs. SD group.

Results from western blot analysis showed that the level of cPKCγ membrane translocation in the REM-SD, REM-SD+PMA, REM-SD+H-7, and SR groups were all significantly lower than that in the control group. Compared with REM-SD group, levels were higher in the SR and REM-SD+PMA groups and lower in the REM-SD+H-7 group (Figure 6).

### REM-SD Reduces Ng and Phosphorylated Ng Expression

**Figure 7:**
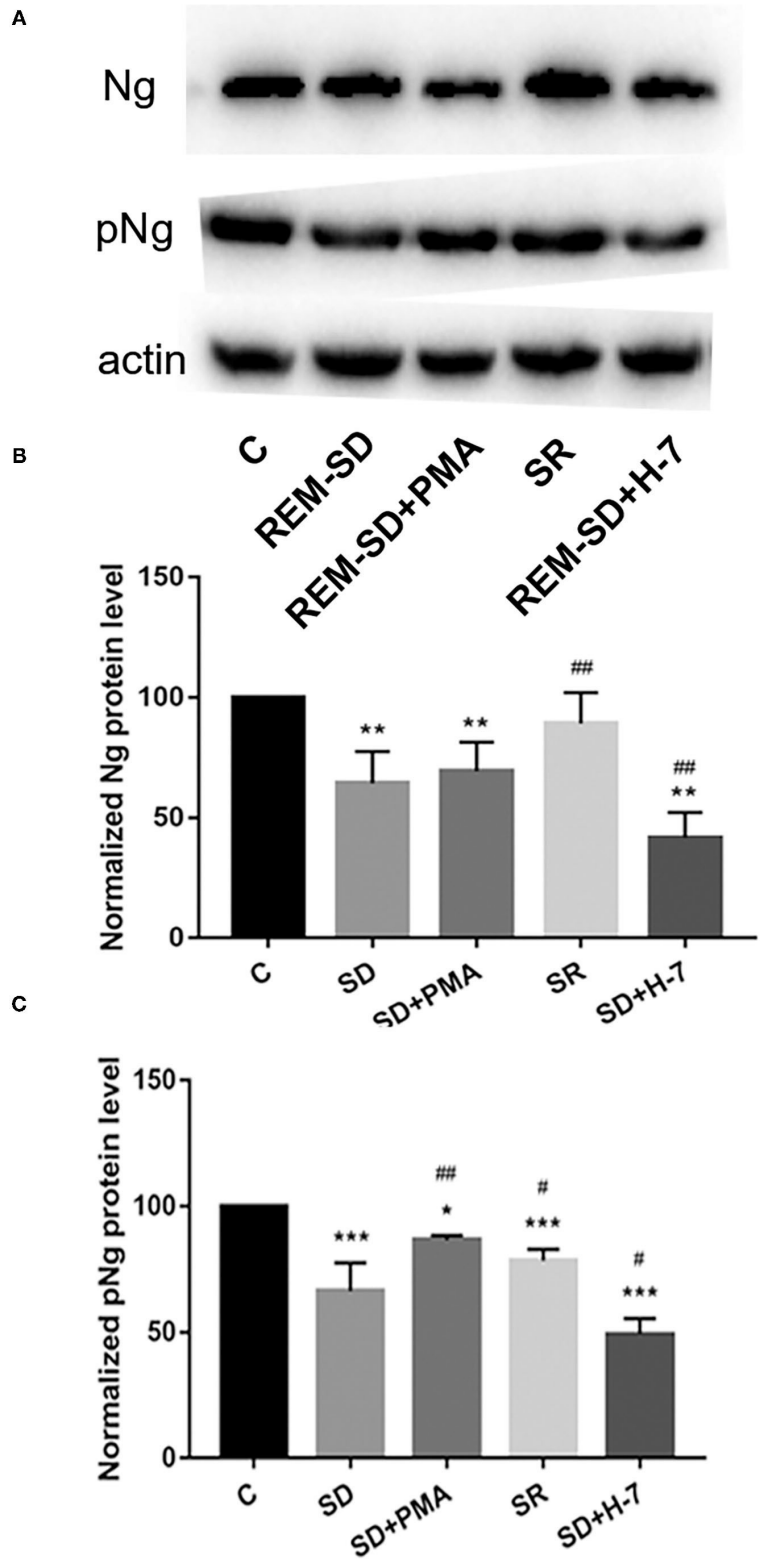
Protein expression for Ng and phosphorylated Ng in the prefrontal cortex of SD rats from each group and control group (C). **(A)** Western blot analysis results. **(B)** Relative protein expression of Ng for each group. **(C)** Relative protein expression of phosphorylated Ng for each group. *P < 0.05, **P < 0.01, ***P < 0.001 vs. control; ^#^*P* < 0.05, ^##^*P* < 0.01 vs. SD group.

Western blot analysis showed that Ng protein expression in REM-SD/ REM-SD+PMA/ REM-SD+H-7 experimental groups were significantly lower than in the control group. Compared with the REM-SD group, Ng protein expression was significantly lower in the REM-SD+H-7 group, tended to be higher in REM-SD+PMA group, and was significantly higher in the SR group (Figure 7).

The expression of phosphorylated Ng in all experimental groups was lower than in the control group. Compared with the REM-SD group, expression of phosphorylated Ng was significantly higher in the SR and REM-SD+PMA groups and was significantly lower in the REM-SD+H-7 group (Figure 7).

## Discussion

In the present study, we confirmed that REM-SD can significantly reduce spatial learning and memory ability in rats. Western blot analysis showed that cPKCγ activity and Ng phosphorylation levels were significantly reduced following REM-SD, which suggests that REM-SD-induced impairments in spatial learning and memory in rats are associated with cPKCγ-Ng signal transduction.

We used a modified multiple platform method to selectively deprive rats of REM sleep. When rats enter REM sleep, they lose their muscle tone and fall into the water, thus waking them up and making rats unable to enter REM sleep. After 72 h of REM-SD, we observed expected symptoms of REM-SD, including increased food intake, lassitude, dullness, lack of luster, dryness, disheveled and untidy fur, increased excitability and irritability, and increased alertness, responsiveness, and aggressive behavior in response to environmental stimuli.

We used the Morris water maze to assess changes in spatial learning and memory. The place navigation test revealed that the average time needed to find the platform (escape latency) was significantly prolonged in the REM-SD group compared with control group, which indicates that REM-SD can significantly impair spatial learning and memory ability in rats. Although escape latency in the SR group was significantly shorter than that of the REM-SD group, it was still longer than that of the control group. This indicates that SR following REM-SD only partially restores learning and memory ability. During the spatial probe test, the percentage of time spent in the target quadrant was significantly shorter in the REM-SD group than that in the control group, which indicates that REM-SD can cause damage to the acquisition and maintenance of spatial memory, which is consistent with previous resultsT. The percentage of time spent in the target quadrant was significantly longer in the SR group compared to the REM-SD group, and was shorter in the SR group than in the control group, which indicates that after 12 h of SR, spatial memory was partially improved. This further validated the results of the place navigation test. We also found that the REM-SD group spent significantly more time in the first quadrant than did the control group, while the SR group spent significantly less time in the first quadrant compared with the REM-SD group, further suggesting that REM-SD can impair spatial learning and memory.

PKC is a family of serine–threonine kinases that is widely expressed in mammalian tissues. PKC is essential for signal transduction involved in sleep/wake regulation and sleep-related behaviors (33–35). Studies focused on cPKCγ have attracted broad attention. Although some reports have found that cPKCγ is involved in learning and memory (19, 20), its role in REM-SD-induced learning and memory impairment is still unknown. In the present study, we found that cPKCγ protein expression was significantly reduced after REM-SD and was increased following SR. However, administration of the cPKCγ activator PMA and the inhibitor H-7 had no effects on cPKCγ protein expression. cPKCγ is present in the cytoplasm in an inactive form when at rest. The regulatory domain interacts with the catalytic domain through a pseudosubstrate sequence and prevents the substrate from entering the catalytic region. cPKCγ is activated upon translocation to the cell membrane under external stimulus conditions (20, 36). We therefore also investigated the degree to which cPKCγ was translocated to the membrane and found that it was markedly lower in the REM-SD group than in the control group. This indicates that REM-SD can significantly inhibit cPKCγ membrane translocation and activation. SR after REM-SD can increase cPKCγ membrane-translocation levels and thus promote cPKCγ activation, although activation levels did not reach pre-SD levels. These results are consistent with the Morris water maze test results. Similarly, administering the cPKCγ activator PMA significantly increased cPKCγ membrane translocation, and thus promoted its activation. In contrast, the cPKCγ inhibitor H-7 significantly reduced cPKCγ membrane translocation and activation. These changes are also consistent with the behavioral results; the REM-SD+PMA group performed better than the REM-SD group on the place navigation and spatial probe tests, whereas the REM-SD+H-7 group performed worse than both untreated and REM-SD rats. These findings indicate that changes in the level of cPKCγ membrane translocation are related to REM-SD-induced learning and memory impairment.

Ng is a natural postsynaptic PKC substrate that is abundantly expressed in brain regions, and which is involved in cognitive function (36). Ng is a key CaM-binding protein that buffers CaM function. Phosphorylation of Ng by PKC at serine 36 (Ser36) reduces its binding to CaM, allowing CaM to bind to calcium and activate CaM-dependent pathways, such as the Ca^2+^/Calmodulin-Dependent Protein Kinase II (CaMKII) signal cascade, which is involved in learning and memory. Zhong et al. revealed the importance of the PKC signaling system for CaM-Ng-dependent synaptic plasticity; the ratio of phosphorylated/non-phosphorylated Ng can determine the CaMKII- associated LTP/LTD threshold (37). Our western blot analysis results showed that REM-SD significantly reduced cPKCγ membrane translocation and Ng phosphorylation levels, which suggests that REM-SD-induced memory impairment is mediated by inhibition of the cPKCγ and Ng signaling-transduction pathway. REM-SD may prevent the buffering effect of non-phosphorylated Ng over CaM, leading to abnormal CaM signaling. Administration of the cPKCγ activator PMA significantly increased Ng phosphorylation levels and the cPKCγ inhibitor H-7 significantly inhibited Ng phosphorylation, which suggests that Ng is downstream of the cPKCγ signaling pathway. The cPKCγ inhibitor H-7 can significantly reduce Ng protein expression and the cPKCγ activator PMA can increase Ng protein expression. Although no statistical difference was found between groups, the results further indicate that cPKCγ not only regulates the activation and phosphorylation of Ng, but can also promote Ng protein expression.

REM sleep plays an important role in the consolidation of long-term memory in the nervous system, which can strengthen the newly-formed connections made during wakefulness, form new connections, and enhance new memories during subsequent wakefulness. Thus, we can hypothesize that the role of the cPKCγ-Ng pathway on REM-SD-induced learning and memory impairment may be associated with it involvement in the establishment of new synaptic connections during the learning and memory consolidation. In future work, we will use neurophysiological methods to perform electrophysiological recordings of the pre-frontal cortex in rats, and investigate the role of cPKCγ-Ng in long-term synaptic plasticity during REM-SD.

There are some limitations to the current study. The main purpose of the modified multiple platform method is used in the present study to investigate REM (30, 31). The EEG results also support this is an effective performance to reduce the 95% of REM (30, 32). However, Alkadhi et al. found that non-REM (NREM) sleep was signficantly reduced by the multiple platform method (38). Therefore, we cannot completely exclude the effects of NREM deprivation on the impairment of spatial lerning and memory. Additionally, it has been reported that expression levels of both Ng mRNA and Ng protein differ across different brain regions after 24 h of SD, and Ng protein levels were significantly reduced in the cortex (29). Based on these findings, we only focused on the pre-frontal cortex. However, the hippocampus is a key area for spatial learning and memory. Further investigations should explore the changes in the PKC-Ng signaling pathway in the hippocampus of rats to confirm our results. Furthermore, due to a lack of specific activators and inhibitors of cPKCγ, we used the non-specific activators and inhibitors of the PKC family, PMA and H-7. Also, it remains unclear without administration of PMA/H-7 will affect the response during water maze test. Administering PMA can promote cPKCγ activation and increase Ng phosphorylation levels, and administering H-7 showed the opposite effect; this result indicates that PMA and H-7 have excitatory and inhibitory effects on cPKCγ, respectively. Other groups' studies have found that many signaling pathways are involved in REM-SD-induced cognitive impairment, including NMDA receptor pathways; 24-h SD impairs NMDAR function in the rat dentate gyrus and 72-h REM-SD also impairs NMDAR function in the rat hippocampal CA1 area (33, 34). Thus, in the future, we will investigate other possible downstream targets of the cPKCγ signaling pathway, or other signaling pathways that could play a role in learning and memory impairment after REM-SD.

In summary, our results showed that the REM-SD can induce learning and memory impairments in rats which are associated with the cPKCγ-Ng signaling pathway. Activation of the cPKCγ-Ng signaling pathway can significantly improve REM-SD-induced learning and memory impairment, which is expected to become a novel molecular target for treatment of REM-SD.

## Data Availability Statement

The original contributions presented in the study are included in the article/Supplementary Material, further inquiries can be directed to the corresponding authors.

## Ethics Statement

All animal experiments were approved by the Ethics Committee of the Medical School of Capital Medical University.

## Author Contributions

SX, YZ, ZX, and LS: contributed significantly to this work, and wrote and revised the manuscript. ZX and LS: designed the research study. SX and YZ: performed the research study and extracted and analyzed the data. All authors approved the final version of the manuscript.

## Funding

This study was supported by the National Science and Technology Support Program in China (2013BAI07B01), the Natural Science Foundation of Shandong Province in China (ZR2012HQ014, ZR2011HM044, ZR2015HL041), and Shandong Province Medical and Health Plan (2014WSB32015), Special Fund of Basic Scientifc Research Service Fee of Central Public Welfare Scientifc Research Institute of China (2015CZ-49, 2019zx-Q9), The National Key Research and Development Program of China (2016YFF0201002).

## Conflict of Interest

The authors declare that the research was conducted in the absence of any commercial or financial relationships that could be construed as a potential conflict of interest.

## Publisher's Note

All claims expressed in this article are solely those of the authors and do not necessarily represent those of their affiliated organizations, or those of the publisher, the editors and the reviewers. Any product that may be evaluated in this article, or claim that may be made by its manufacturer, is not guaranteed or endorsed by the publisher.
