# Corrigendum: Effect of the cPKCγ-Ng Signaling System on Rapid Eye Movement Sleep Deprivation-Induced Learning and Memory Impairment in Rats

**DOI:** 10.3389/fpsyt.2021.824725

**Published:** 2021-12-21

**Authors:** Shu Xu, Yanbo Zhang, Zhiqing Xu, LuPing Song

**Affiliations:** ^1^School of Rehabilitation, Capital Medical University, Neurorehabilitation Center, Beijing Boai Hospital, China Rehabilitation Research Center, Beijing, China; ^2^Department of Neurobiology, The Second Affiliation Hospital of Shandong First Medical University, Tai'an, China; ^3^Department of Neurobiology, Beijing Key Laboratory of Neural Regeneration and Repair, Capital Medical University, Beijing, China; ^4^Department of Rehabilitation Medicine, Shenzhen University General Hospital, Shenzhen, China

**Keywords:** REM sleep deprivation, learning and memory, cPKCγ, neurogranin, signaling

In the published article, there was an error in affiliation **1**. Instead of “**Neurorehabilitation Center, Beijing Boai Hospital, China Rehabilitation Research Center, Beijing, China**,” it should be “**School of Rehabilitation, Capital Medical University, Neurorehabilitation Center, Beijing Boai Hospital, China Rehabilitation Research Center, Beijing, China**.”

The authors apologize for this error and state that this does not change the scientific conclusions of the article in any way. The original article has been updated.

## Publisher's Note

All claims expressed in this article are solely those of the authors and do not necessarily represent those of their affiliated organizations, or those of the publisher, the editors and the reviewers. Any product that may be evaluated in this article, or claim that may be made by its manufacturer, is not guaranteed or endorsed by the publisher.

