# Supplementary figures and images for: Effect of the cPKCγ-Ng Signaling System on Rapid Eye Movement Sleep Deprivation-Induced Learning and Memory Impairment in Rats
Source: Front Psychiatry. 2021 Oct 29;12:763032. doi: 10.3389/fpsyt.2021.763032 (PMC8586205; doi:10.3389/fpsyt.2021.763032)

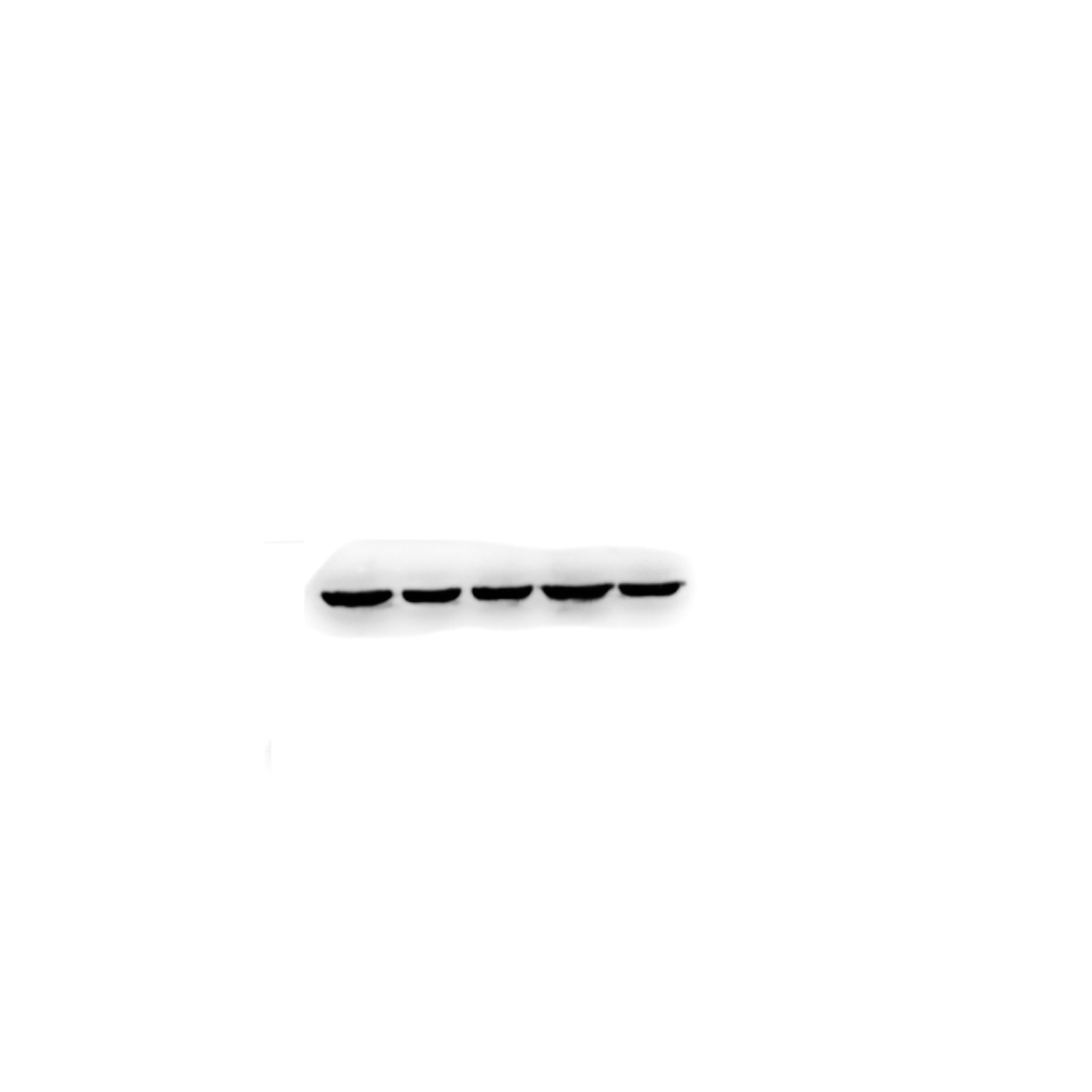

Supplement: Supplementary file 1 [file Image_1.tif]

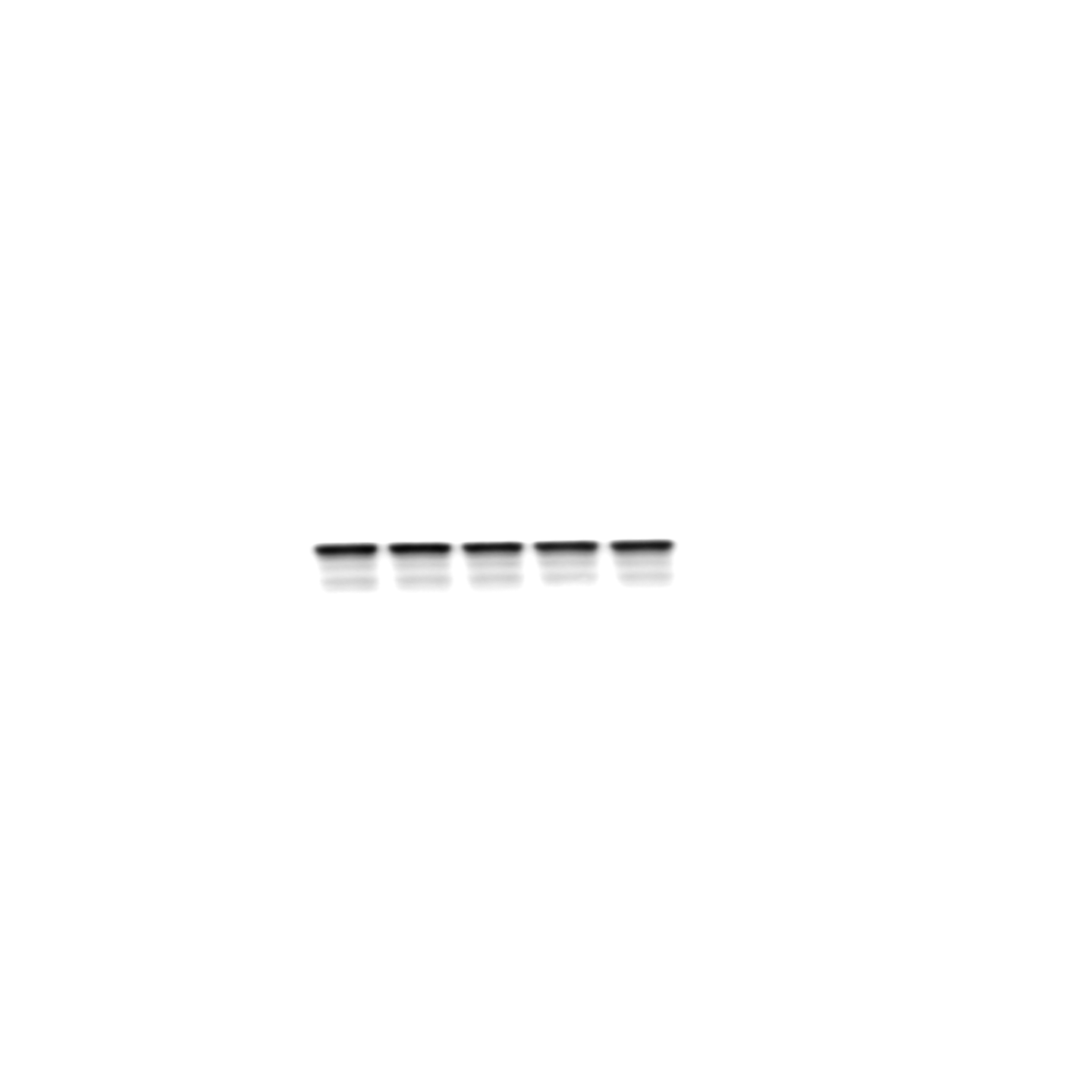

Supplement: Supplementary file 2 [file Image_2.tif]

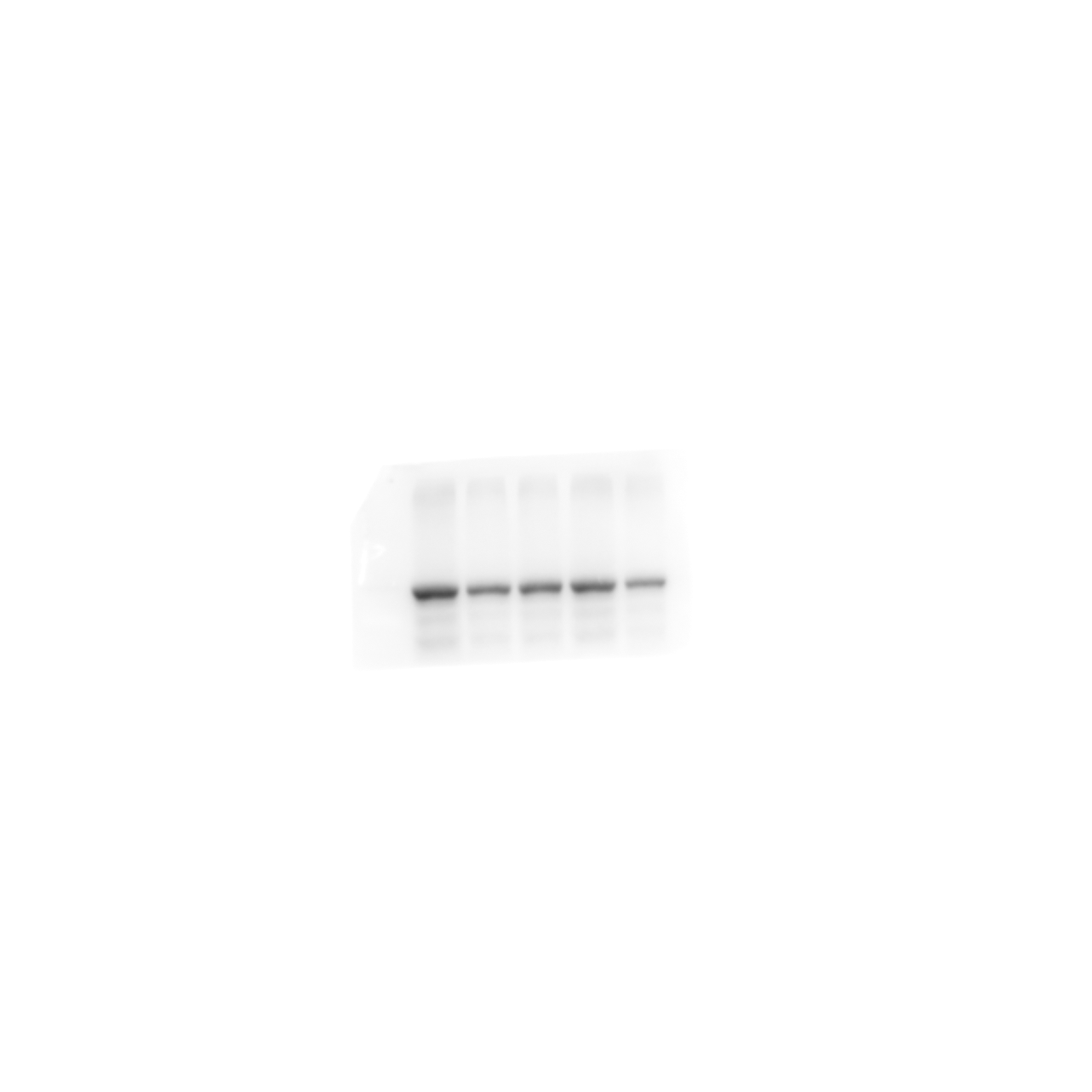

Supplement: Supplementary file 3 [file Image_3.tif]

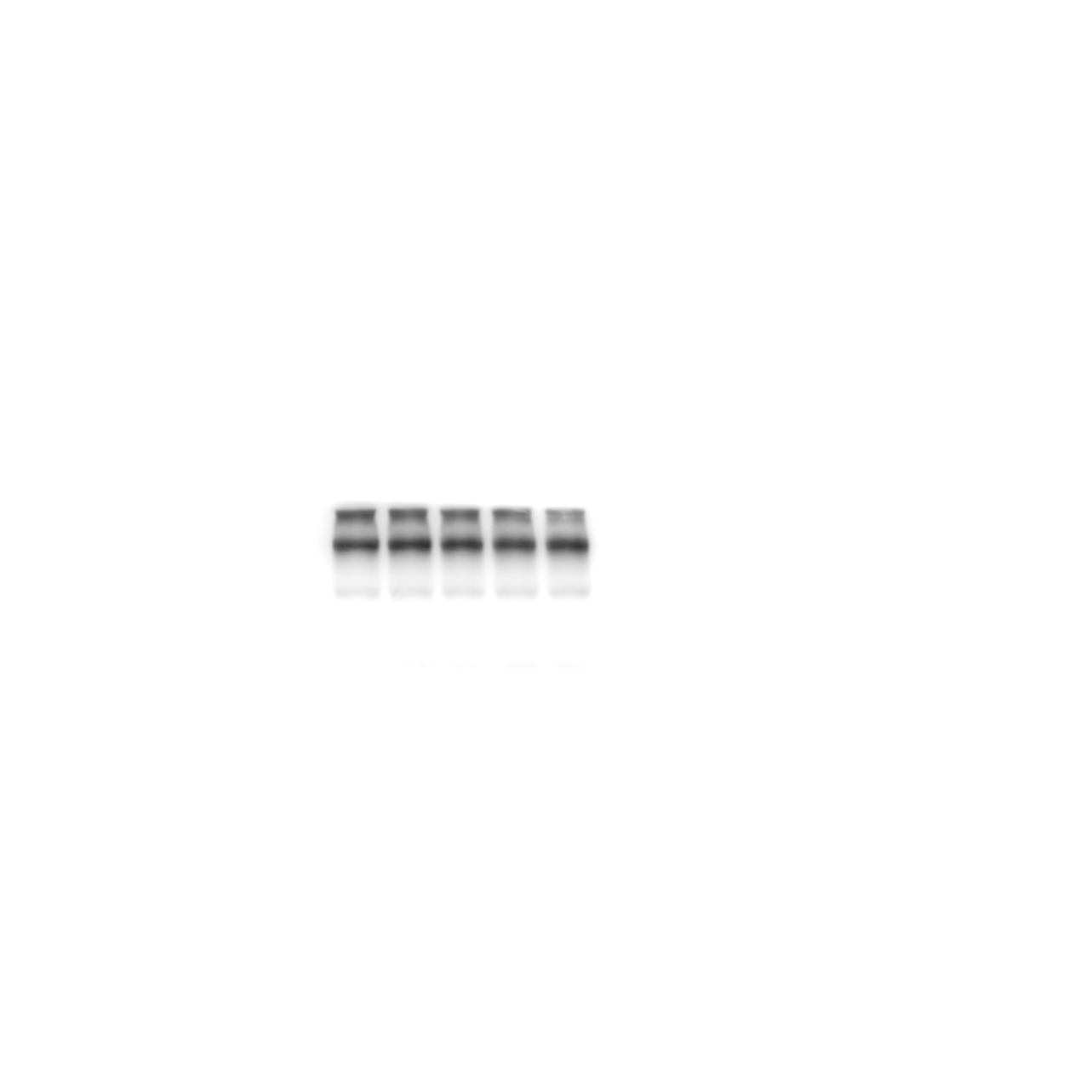

Supplement: Supplementary file 4 [file Image_4.tif]

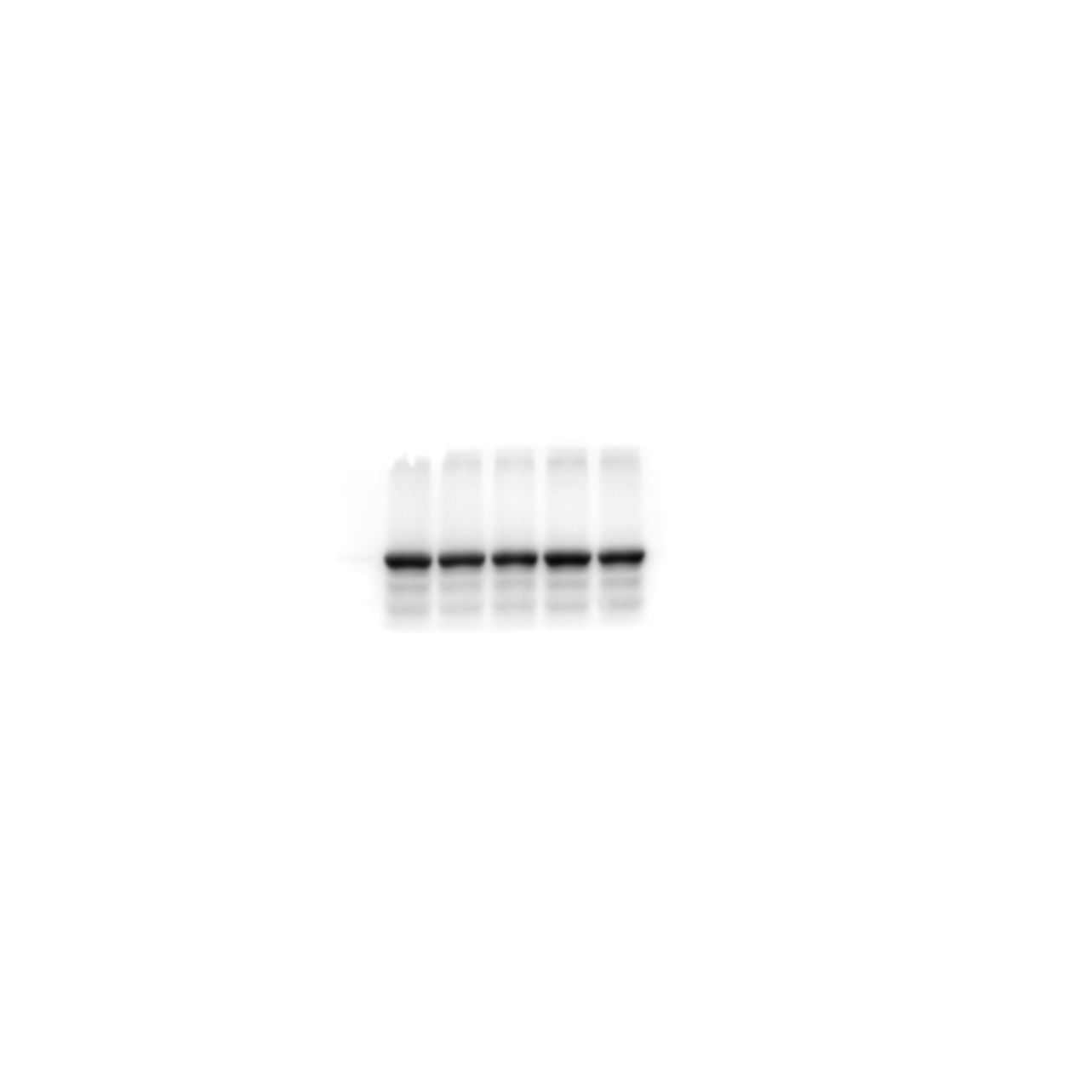

Supplement: Supplementary file 5 [file Image_5.tif]

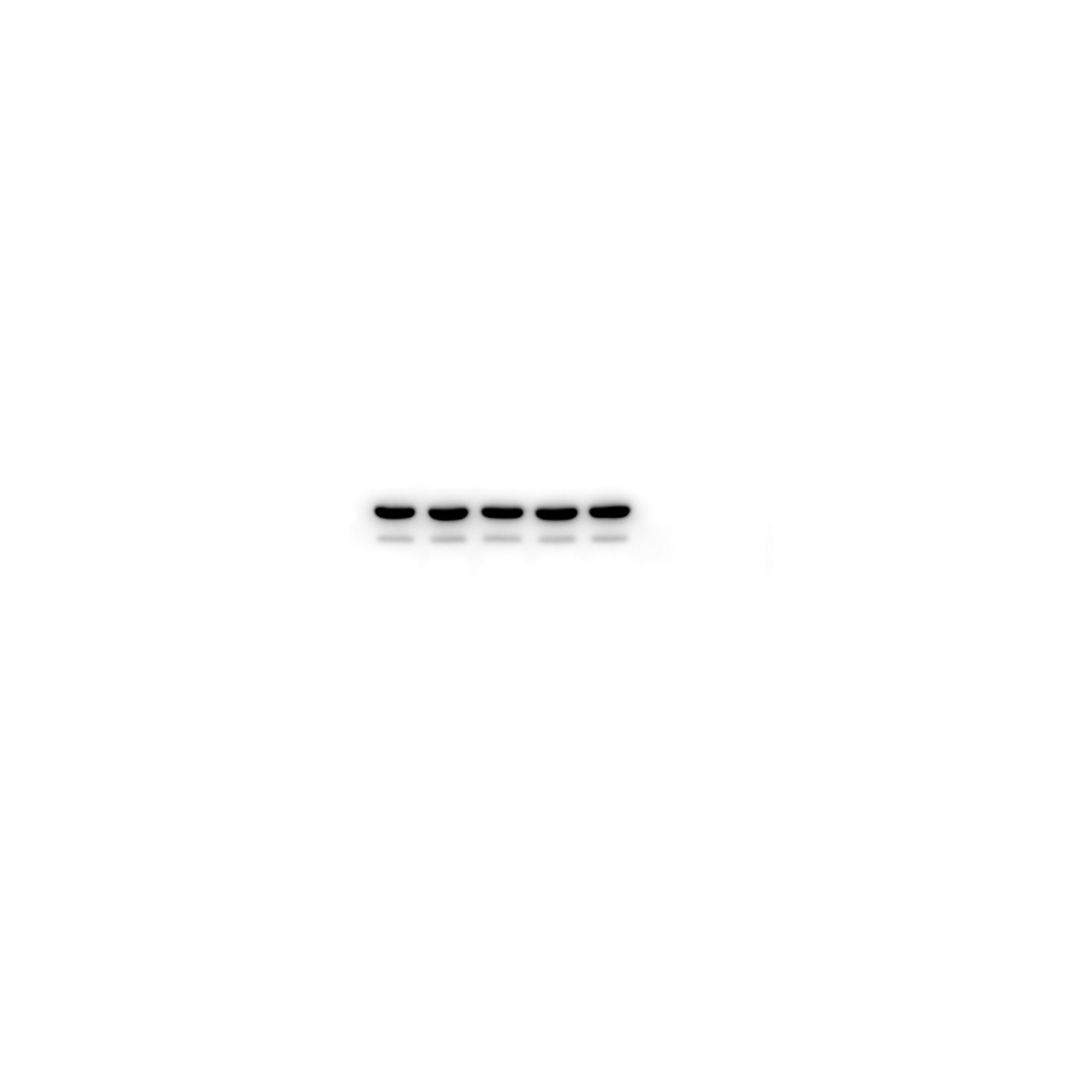

Supplement: Supplementary file 6 [file Image_6.tif]

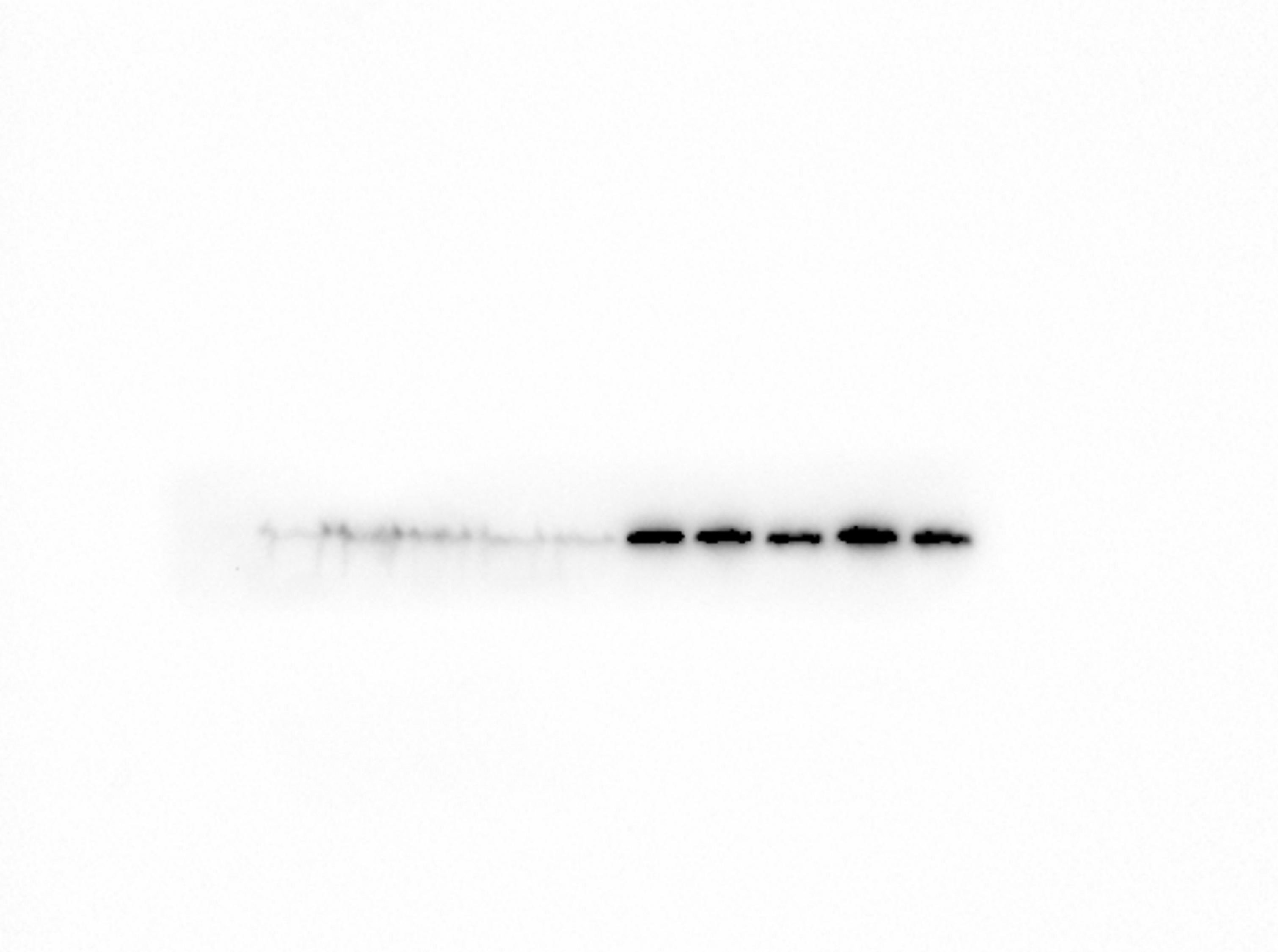

Supplement: Supplementary file 7 [file Image_7.tif]

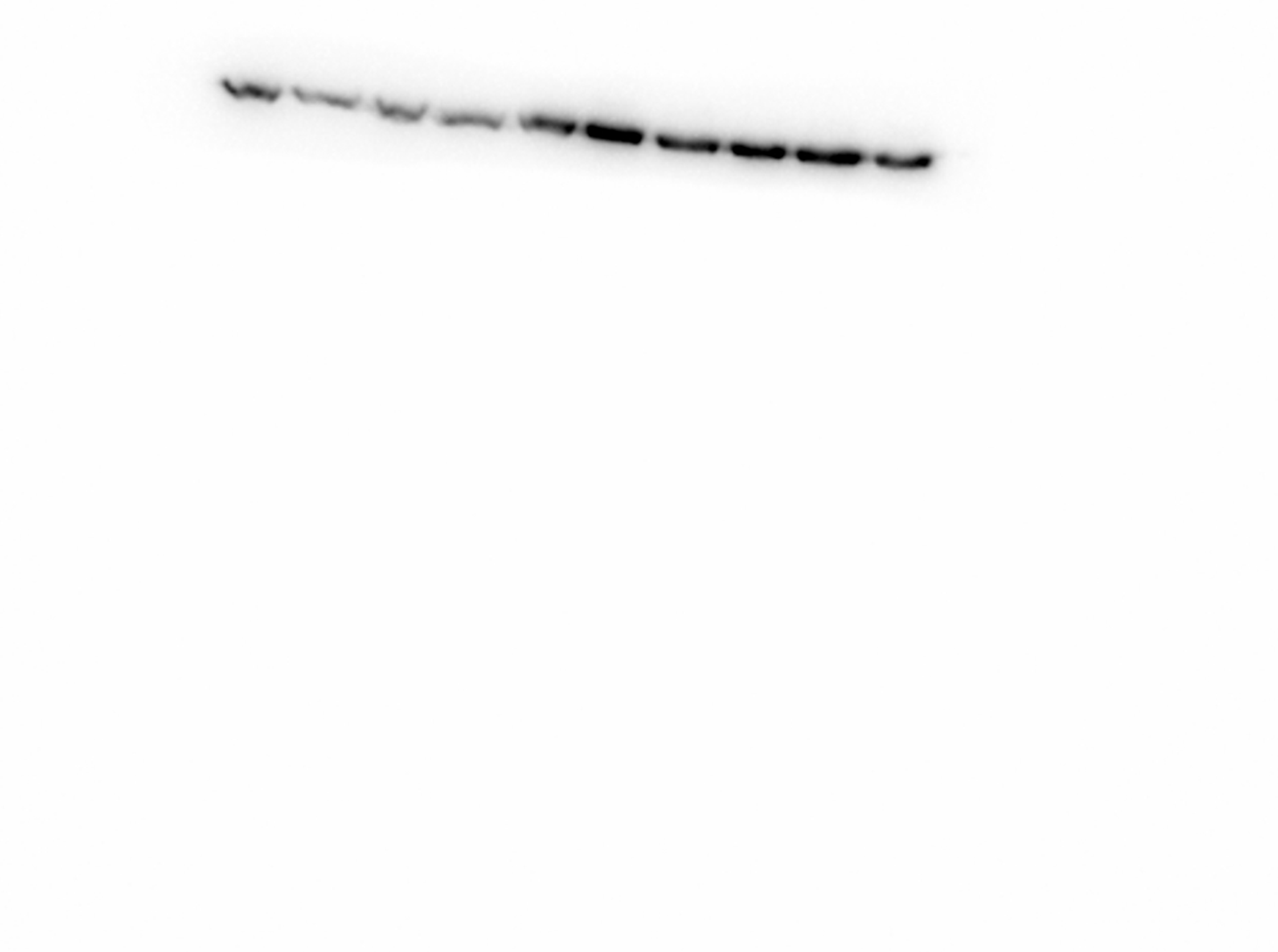

Supplement: Supplementary file 8 [file Image_8.tif]

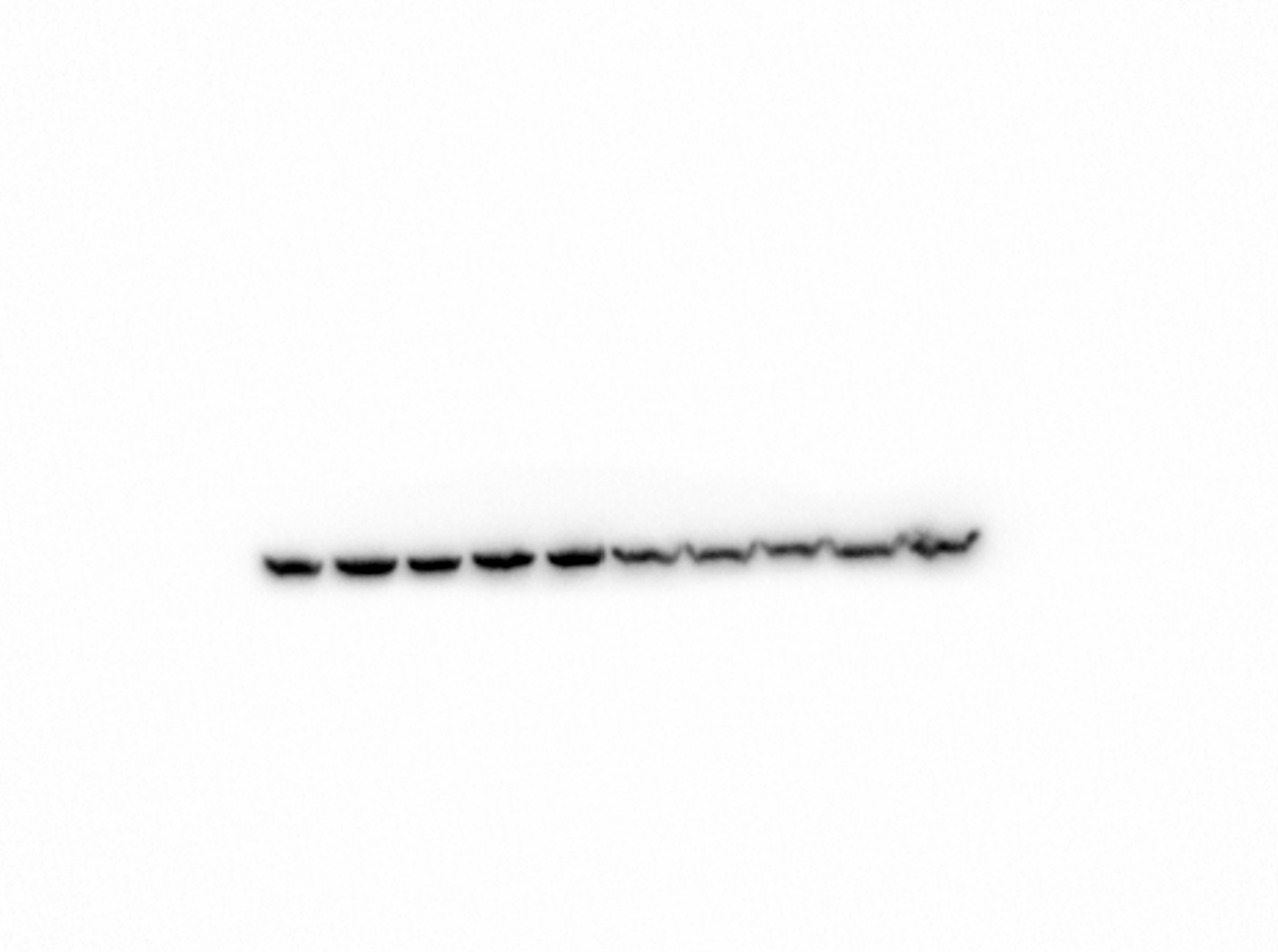

Supplement: Supplementary file 9 [file Image_9.tif]
